# Morphological and physiological changes induced by *Achyranthes aspera*-mediated silver nanocomposites in *Aedes aegypti* larvae

**DOI:** 10.3389/fphys.2022.1031285

**Published:** 2022-10-12

**Authors:** Aarti Sharma, Monika Mishra, Vinay Singh Dagar, Sarita Kumar

**Affiliations:** Insect Pest and Vector Laboratory, Department of Zoology, Acharya Narendra Dev College, University of Delhi, New Delhi, India

**Keywords:** *Achyranthes aspera*, *Aedes aegypti*, development, histological architecture, morphology, silver nanocomposites, stomach poison, TEM

## Abstract

*Aedes aegypti* is responsible for the global spread of several ailments such as chikungunya, dengue, yellow fever, and Zika. The use of synthetic chemicals is the primary intervention in mosquito management. However, their excessive utilization resulting in the spread of toxic ingredients in the environment and posing threats to beneficial organisms has prompted the recommendation for the use of biologically synthesized nanocomposites as a promising approach for vector control. Silver nanocomposites were synthesized using leaf (AL-AgNCs) and stem (AS-AgNCs) extracts of *Achyranthes aspera*. The early fourth instars of *A. aegypti* were exposed to lethal doses of these nanocomposites to evaluate their effects on larval development, behavior, morphology, and mid-gut histoarchitecture. The cellular damage and deposition of nanocomposite residues in the mid-gut were studied using light and transmission electron microscopy. The *A. aspera* silver nanocomposite (AA-AgNC)-exposed larvae exhibited dose-dependent extended duration of development and diminished adult emergence, but did not exhibit modified behavior. Intense damage to the cuticle membrane and slight contraction in the internal membrane of anal papillae were noticed. Morphologically, the mid-gut appeared disorganized, darkly pigmented, and shrunk. Histological investigations of the mid-gut revealed significantly disordered internal architecture with lysed cells, damaged peritrophic membrane and microvilli, disintegrated epithelial layer, and a ruptured and displaced basement membrane. Visualization of the larval mid-gut through TEM showed severe cellular damage and aggregation of black spots, indicating the deposition of silver particles released by AA-AgNCs. The investigations revealed the bio-efficacy of *A. aspera-*mediated AgNCs against *A. aegypti* inducing stomach and contact toxicity in the larvae. The utilization of AA-AgNCs is recommended for *A. aegypti* management as a safe and effective intervention.

## Introduction

*Aedes aegypti* L., the prime and highly widespread mosquito vector, is responsible for the transmission of viruses causing various ailments, such as dengue, Zika, yellow fever, and chikungunya. These diseases are more prevalent in subtropical and tropics regions of the world, although the severity and risks involved are under the influence of climatic, social, and environmental factors. In the last two decades, the incidences of dengue have amplified over 8-fold. In 2000, a total of 505,430 dengue cases were recorded which increased to more than 2.4 million in 2010, reaching 5.2 million in 2019 (World Health Organization, 2022). Nevertheless, the total number of cases and deaths apparently declined during the years 2020 and 2021 which might be due to the under-reporting of cases during the COVID-19 pandemic (World Health Organization, 2022).

In India, dengue fever is pervasive in nearly every single region. The disease has been reported from 36 states/union territories with a total of 44,585 dengue incidences and 56 fatalities in 2020, rising to 193,245 incidences and 346 deaths in 2021 (NVBDCP, 2022). The maximum outbreak was documented in Uttar Pradesh (29,750 cases) followed by Punjab and Rajasthan. Likewise, India encountered a total of 119,070 chikungunya cases in 2021, the highest in the last few years (NVBDCP, 2022).

Mosquito-borne diseases can currently be kept under check only through mosquito control by employing various interventions among which the main control tool is the application of synthetic insecticides in the form of space sprays, indoor residual sprays (IRS), insecticide-treated nets (ITNs), and long-lasting insecticide-treated nets (LLINs) (Dulacha et al., 2022). These measures have not only created serious health and environmental issues but also made mosquitoes resistant to insecticides, forcing the use of higher doses of chemicals at a higher frequency. These issues have severely impacted the survival of non-target organisms and disturbed our ecosystem, specifically due to the bio-magnification of these toxicants in the food chain. Thus, the identification of effective and eco-friendly control methods is necessitated, which can be used safely and effectively.

In recent years, metallic nanoparticles formulated with diverse metals possessing infrequent and varied physical and chemical properties have engrossed substantial interest among researchers (Sabri et al., 2016). Several kinds of nano-formulations have been prepared using various agents, especially plants and microbes, which have emerged as probable bio-pesticides. Thus, attempts have been made to formulate metallic nanoparticles by employing different metals, such as iron, zinc, silver, gold, platinum, and titanium. Among these, silver nanoparticles have been investigated at a larger scale and reported to exhibit toxic potential against bacteria, fungi, and mosquito larvae (Ga’al et al., 2018). The formulation of these composites using phytoconstituents is considered a highly advantageous and rapid approach in comparison to their fabrication using chemical or physical methods. This is because phytomediated measures do not require any toxicants, high temperature, energy, pressure, and purification steps; phytoconstituents can act as reducing agents and capping agents and control the biophysical parameters of nanoparticles (Govindarajan et al., 2016; Sabri et al., 2016).

Various biological agents have been studied for a few years in an effort to produce inexpensive, facile, and effective metallic nanoparticles against mosquitoes (Thakkar et al., 2010; Salunkhe et al., 2011; Tran and Le, 2013; Shah et al., 2015; Ahmed et al., 2016). Silver nanoparticles have been synthesized using many plants, such as extracts of neem (*Azadirachta indica*), green tea (*Camellia sinensis*), natural rubber, *Aloe vera*, and lemongrass (*Cymbopogon citratus*) (Vijayaraghavan et al., 2012); *Ambrosia arborescens* leaf extract (Morejón et al., 2018); and *Annona reticulata* leaf extract (Parthiban et al., 2019). Various plants used in the formulation of silver nanoparticles and possessing toxic activity against larvae of different mosquito species have been reviewed and documented (Kabtiyal et al., 2022). Apart from this, the impact of silver nanoparticles prepared from *Hedychium coronarium* (Kalimuthu et al., 2017) and *Cymodocea serrulata* (Amutha et al., 2019) aqueous extracts has also been evaluated on the mid-gut histoarchitecture of the *A. aegypti*, *Culex quinquefasciatus*, and *Anopheles stephensi* larvae.

The leaf, flower, and seed extracts of a perennial herb *A. aspera* have been reported to exhibit biological activity against *A. aegypti*, *A. subpictus*, and *C. tritaeniorhynchus*, having diverse effects on each development stage (Bagavan et al., 2008). The plant has been used to treat malaria, pneumonia, cough, asthma, snake bites, stomach aches, skin eruptions, boils, and heaps (Amaladhas et al., 2013; Elumalai et al., 2016; Sharma et al., 2016). The larvicidal efficacy of silver nanoparticles synthesized from the leaf and stem extracts of *A. aspera* has been assessed against *A. aegypti*, revealing respective LC_50_ values of 5.819 and 9.119 μg/ml after 24 h (Sharma et al., 2017; Sharma et al., 2020). The current study estimated the impact of these nanocomposites on the morphology, behavior, and development of the early fourth instar of *A. aegypti.* The effect of the nanocomposites on the mid-gut cellular architecture of larvae was evaluated through light microscopy, ultra-histology, and transmission electron microscopy. It is believed that the outcomes of the study would assist in safe and effective mosquito management.

## Materials and methods

The investigations were carried out with silver nanocomposites prepared from *A. aspera* leaf and stem extracts against the early fourth instars of a laboratory-reared strain of *A. aegypti.* The larvae were exposed to pre-determined lethal dosages of each nanocomposite for 24 h. The effect of nanocomposites on the behavior, morphology, growth, and development of larvae was assessed. The toxic impact on the mid-gut histological architecture of larvae was also studied.

### Rearing of *A. aegypti*

The dengue vector, *A. aegypti*, used in the current investigations, has been maintained in a dedicated laboratory at Acharya Narendra Dev College, University of Delhi, India. Rearing is carried out under the controlled condition of relative humidity (75 ± 10%), temperature (27 ± 2°C), and photoperiod (14 L:10 D). Adults are kept in a muslin cloth cage and fed sugar syrup of water-soaked deseeded raisins. The females, provided with blood meals on alternate days for egg maturation, lay eggs in a water-filled bowl lined with strips of Whatman filter paper No. 1. The eggs are hatched in a plastic or enamel tray half-filled with de-chlorinated water. The larvae are provided dog biscuits and yeast (3:2 w/w) for nutrition and are maintained carefully. The pupae were separated out and kept in the screened cloth cages for adult emergence.

### Preparation of *A. aspera* extract

The fresh leaves and stems of *A. aspera* were gathered from the areas surrounding the institute. The plant was identified by experts in the Department of Botany, University of Delhi, India, and a sample was submitted in their herbarium with accession no. 14376. The collected parts were washed thoroughly and cut into small pieces. The 10 g pieces of each part were added to 100 ml of double-distilled water and heated at 60°C for 15–20 min. The aqueous extracts formed were left undisturbed for 3 h and then filtered to obtain a clear solution (Sharma et al., 2017).

### Formulation of *A. aspera* silver nanocomposites

The silver nanocomposites were synthesized from the leaf and stem aqueous extracts of *A. aspera* following a standardized protocol used in our earlier studies (Sharma et al., 2017; Sharma et al., 2020). The 15 ml *A. aspera* leaf extract (AALE) and 18 ml *A. aspera* stem extract (AASE) were separately added to 100 ml of 4 mM silver nitrate solution. The silver nitrate–AALE mixture was incubated at room temperature in the dark for 24 h, while that of the AASE was first boiled at 85–90°C in a water bath for 30 min. The alteration in the color of the solution was recorded to confirm the synthesis of silver nanocomposites which were subsequently incubated in the dark for 24 h and centrifuged at 12,000 rpm for 10–15 min. The supernatant was collected and re-centrifuged, resulting in the formation of powdery pellets which were stored at room temperature for conducting bioassays.

### Effect of *A. aspera* silver nanocomposites on larval growth and development

Our earlier studies have estimated the LC_50_ values of AgNCs formulated with leaf (AL-AgNCs) and stem (AS-AgNCs) extracts of *A. aspera* (Sharma et al., 2017; Sharma et al., 2020). The respective LC_30_, LC_50_, and LC_90_ values obtained with exposure of *A. aegypti* larvae to AL-AgNCs were recorded as 1.681, 5.819, and 21.068 μg/ml, and with AS-AgNC exposure as 1.750, 9.119, and 15.48 μg/ml. The effect of all these dosages on the growth and development of *A. aegypti* larvae was estimated. A total of 20 larvae were exposed to individual dosages for 24 h. The larvae which survived were monitored until adult emergence. The developmental period and adult emergence were recorded to assess the latent effects of NCs. The studies were conducted in three replicates with each dosage.

### Effect of *A. aspera* silver nanocomposites on larval behavior

A total of 20 early fourth instars of *A. aegypti* were exposed to median lethal doses of leaf and stem AA-AgNCs, separately. The larvae were observed at specific time intervals of 1, 3, 6, and 9 h, followed by final observations at 24 h. The changes in their behavior pattern such as wriggling, swiftness, excitation, agitation, aggression, aggregation, horizontal or vertical movements, inability to rise up to the water surface, unnatural positions, uncertain movements, and convulsions were documented. The study was conducted with each dosage in three replicates.

### Effect of *A. aspera* silver nanocomposites on larval morphology

The *A. aegypti* early fourth instars were exposed to the median lethal dosage of leaf and stem AA-AgNCs for 24 h, 20 larvae in three replicates. The dead larvae were examined carefully under a zoom stereo binocular microscope (Magnus; MSZ-B1) for morphological alterations. The changes in the head, thorax, abdomen, and external organs, such as the eyes, antennae, mouth brushes, saddle, setae, siphon, and anal gills, were scrutinized. The larvae were also observed critically for changes in their body pigmentation or any other visible aberrations. The observed morphological features in the AA-AgNC-exposed larvae were compared with those of the controls and assessed.

### Effect of *A. aspera* silver nanocomposites on the larval mid-gut histological architecture

The effect of LC_50_ dosages of *A. aspera* silver nanocomposites on the mid-gut histological architecture of 24 h-exposed *A. aegypti* early fourth instars was also evaluated. Control sets were exposed to the aqueous solution of AgNO_3_. The studies were conducted using light microscopy, ultra-histology, and transmission electron microscopy.

### Light microscopy

Post-exposure, the middle body portion of 3–5 moribund larvae was excised of their anterior and posterior portions and fixed in 10% neutral buffered formalin for 24 h. The fixed larvae were washed thoroughly under running water and dehydrated by passing through the ascending series of alcohol grades, 30%, 50%, 70%, 90%, and 100% alcohol (Merck), for 60 min in each. The dehydrated larvae were treated with acetone for 30 min and xylene solution for 2 h. These larvae were embedded in paraffin wax at 55°C for overnight infiltration.

The 4–6 μm thick longitudinal sections of the larval mid-gut were cut using a microtome (Weswox Optik). The sections were made free of wrinkles/folds by treating them with warm water (40–50°C) and dried on albumin-smeared microscope glass slides. The sections were de-waxed in xylene solution for 30 min, followed by one change in acetone and hydration in the descending series of degraded alcohol (100%, 90%, 70%, 50%, and 30%) and distilled water. The hydrated sections were stained with hematoxylin stain and dehydrated again until 90% alcohol concentration. These sections were now counter-stained with eosin for 2–3 min, following which they were kept in absolute alcohol followed by two changes in absolute alcohol: xylene (1:1) for 10 min each. The sections were mounted on a slide with a drop of DPX mountant. The mid-gut tissues were observed critically under a Nikon light microscope (E600) at 40X, examined for alterations relative to the control, and photographed using a Canon Power Shot Digital Camera (SX50HS).

### Ultra-thin histology and transmission electron microscopy

The NC-exposed moribund larvae were fixed in Karnovsky’s fixative (4% paraformaldehyde and 1% glutaraldehyde solution in 0.1 M of phosphate buffer; pH = 7.4) at 4°C for 24 h. Subsequently, they were washed thrice with 0.1 M phosphate buffered saline (PBS) for 15–30 min at 4°C. After fixation, the larvae were treated with 1% (w/v) osmium tetroxide for 15 min at 4°C followed by 3× washing again with PBS at 4°C. Each larva was dehydrated at room temperature by keeping it in the ascending graded series of acetone for 15 min each, treated with absolute alcohol twice for 30 min each, and cleared with toluene twice for 15–20 min. Infiltration was carried out by placing the sample for 2 h in each toluene and raisin mixture (3:1, 2:2, and 1:3), one by one, followed by keeping in pure raisin for 2 h.

The embedded tissue was longitudinally sectioned at 0.5 μm thickness (for slide preparation) and 70 nm thickness (for copper grid preparation) using ultra-microtome (Leica EM UC6). The sections were stained with toluidine blue, observed meticulously, and photographed using a transmission electron microscope (TECNAI 200 Kv ) (Fei, Electron Optics).

## Results

The current investigations used early fourth instar larvae of *A. aegypti* reared in the laboratory and exposed to lethal dosages of leaf and stem AA-AgNCs for 24 h. The exposure effect on the growth, development, behavior, morphology, and mid-gut histological structure of larvae was assessed.

### Effect of *A. aspera* silver nanocomposites on larval growth and development

The larvae exposed to sub-lethal, median-lethal, and lethal dosages of AA-AgNCs showed delayed development and reduced adult emergence in a dose-dependent manner. The development duration from larvae to adults in control sets was 6.00 days which increased significantly by 4.0, 7.0, and 8.67% on exposure to LC_30_, LC_50_, and LC_90_ dosages (*p* < 0.05), respectively, of *A. aspera* leaf nanocomposites (Table 1). The more intense delaying effects on the development duration were imparted by AS-AgNCs, which increased the development duration of larvae by 6.0, 9.0, and 11.66% at LC_30_, LC_50_, and LC_90_ dosages, respectively, compared to controls (Table 2).

**TABLE 1:** Development duration and the adults emerged from the early fourth instar larvae of *Aedes aegypti* exposed to the silver nanocomposites synthesized from the leaf extracts of *Achyranthes aspera* (AL-AgNCs) for 24 h.

Exposure dosage of AL-AgNCs	Development duration of larva to adult (in days) ± SEM	% Relative change in duration w.r.t. control	Total adult emergence ± SEM	% Adult emergence
Control	6.33 ± 0.333a	—	19.00 ± 1.000a_1_	95.00
LC_30_ (1.681 μg/ml)	10.333 ± 0.881b	(+) 4.0	9.00 ± 1.000b_1_	45.00
LC_50_ (5.819 μg/ml)	13.333 ± 0.577c	(+) 7.0	7.00 ± 1.154b_1_	35.00
LC_90_ (21.068 μg/ml)	15.00 ± 0.577c	(+) 8.67	2.66 ± 0.333c_1_	13.33

Mean ± SEM (standard error of mean) calculated for a total of three replicates each comprising 20 larvae. Means in each column followed by different letters are significantly different (*p* < 0.05); one-way ANOVA followed by Tukey’s all-pairwise multiple-comparison test.

**TABLE 2:** Development duration and the adults emerged from the early fourth instar larvae of *Aedes aegypti* exposed to the silver nanocomposites synthesized from the stem extracts of *Achyranthes aspera* (AS-AgNCs) for 24 h.

Exposure dosage of AS-AgNCs	Development duration of larva to adult (in days) ± SEM	% Relative change in duration w.r.t. control	Total adult emergence ± SEM	% Adult emergence
Control	6.00 ± 0.577a	—	19.66 ± 0.333a_1_	98.33
LC_30_ (1.750 μg/ml)	12.00 ± 0.577b	(+) 6.0	8.33 ± 0.881b_1_	41.66
LC_50_ (9.119 μg/ml)	15.00 ± 0.577c	(+) 9.0	5.00 ± 0.577c_1_	25.00
LC_90_ (15.48 μg/ml)	17.66 ± 0.333d	(+) 11.66	2.33 ± 0.333c_1_	11.66

Mean ± SEM (standard error of mean) calculated for a total of three replicates each comprising 20 larvae. Means in each column followed by different letters are significantly different (*p* < 0.05); one-way ANOVA followed by Tukey’s all-pairwise multiple-comparison test.

Likewise, a significant and dose-dependent effect of AA-AgNCs (*p* < 0.05) was recorded on the number of adults that emerged from the exposed larvae indicating the latent effects of formulations. Compared to 95–98.33% of adults which emerged in the control sets, just 13.33–11.66% adults emerged on AA-AgNC exposure at the LC_90_ dosage (Tables 1, 2). Even other dosages negatively impacted emergence at a significant level; however, greater effects were imparted by AS-AgNCs.

### Effect of *A. aspera* silver nanocomposites on the larval behavior

The 24 h exposure with LC_50_ values of leaf and stem AA-AgNCs could not induce any behavioral modifications in the early fourth instars of *A. aegypti*. The larvae showed normal wriggling movements without any restlessness or excitation. Nevertheless, larval mortality of 48.33% and 51.66% was recorded with AL-AgNCs and AS-AgNCs, respectively (Table 3).

**TABLE 3:** Impact of the silver nanocomposites synthesized from leaf and stem extracts of *Achyranthes aspera* on the behavior of early fourth instars of *Aedes aegypti*.

Duration of exposure	AL-AgNCs	AS-AgNCs
LC_50_ dosage	5.819 μg/ml	9.119 μg/ml
00 min	All larvae were active	
01 h	All larvae were active with normal wriggling movements	
3 h	No change in the behavioral pattern, no restlessness, and no excitation	
6 h	No change in the behavioral pattern	
9 h	No mortality and no change in behavioral pattern	
24 h	Mortality %	
	48.33 ± 0.881^a^	51.66 ± 0.333

AL-AgNCs, *Achyranthes aspera* leaf silver nanocomposites; AS-AgNCs, *Achyranthes aspera* stem silver nanocomposites.

^a^ Mean ± SEM, calculated for a total of three replicates each comprising 20 larvae each.

### Effect of *A. aspera* silver nanocomposites on the larval morphology

**FIGURE 1:**
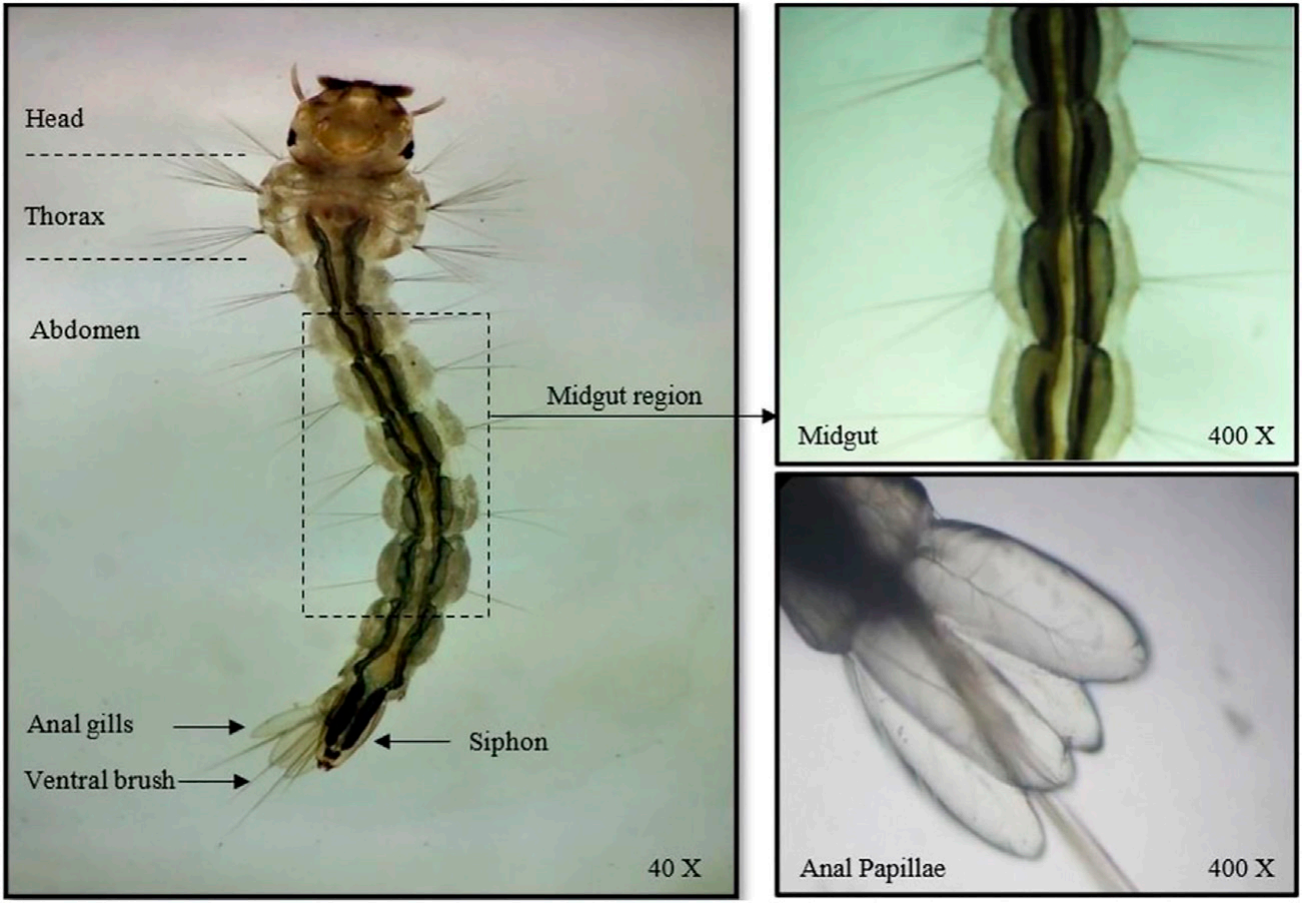
Light photomicrograph of unexposed early fourth instars of *Aedes aegypti* with an enlarged view of the mid-gut and anal papillae.

Observation of the early fourth instars of *A. aegypti* exposed to *A. aspera* leaf and stem mediated-AgNCs under a light microscope revealed no aberrations in their external organs. The larval cuticle, eyes, antennae, mouth, setae, and ventral brushes were normally shaped, similar to that observed in the control larvae (Figure 1).

**FIGURE 2:**
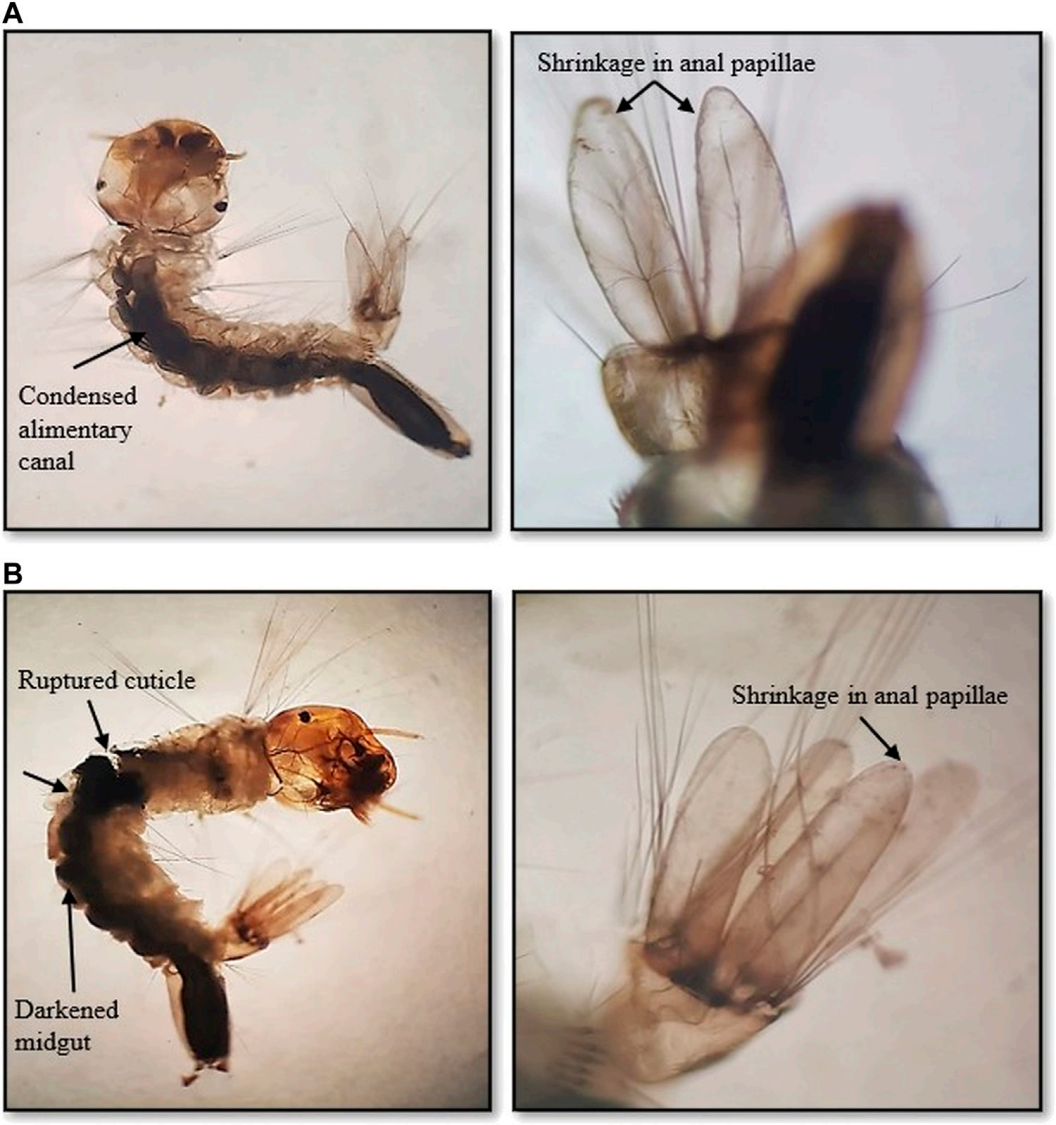
Light photomicrographs of early fourth instars of *Aedes aegypti* with an enlarged view of mid-gut and anal papillae on exposure to **(A)** *Achyranthes aspera* leaf extract-mediated silver nanocomposites (AL-AgNCs) and **(B)** *Achyranthes aspera* stem extract-mediated silver nanocomposites (AS-AgNCs) (→) indicates damaged area showing high pigmentation and blackening of the mid-gut region, ruptured cuticle, and deformed body with shrinkage in anal papillae.

Nevertheless, increased pigmentation and blackening of the gut was observed in the AA-AgNC-exposed larvae (Figures 2A,B). The larval body was shrunk with impaired, destroyed, and disorganized gut portions. In addition, distinct and severe damage in the body cuticular membrane was recorded along with slight shrinkage in the internal membrane of anal papillae. A more pronounced effect was observed when the larvae were exposed to AS-AgNCs (Figure 2B).

### Effect of *A. aspera* silver nanocomposites on the larval mid-gut histological architecture

**FIGURE 3:**
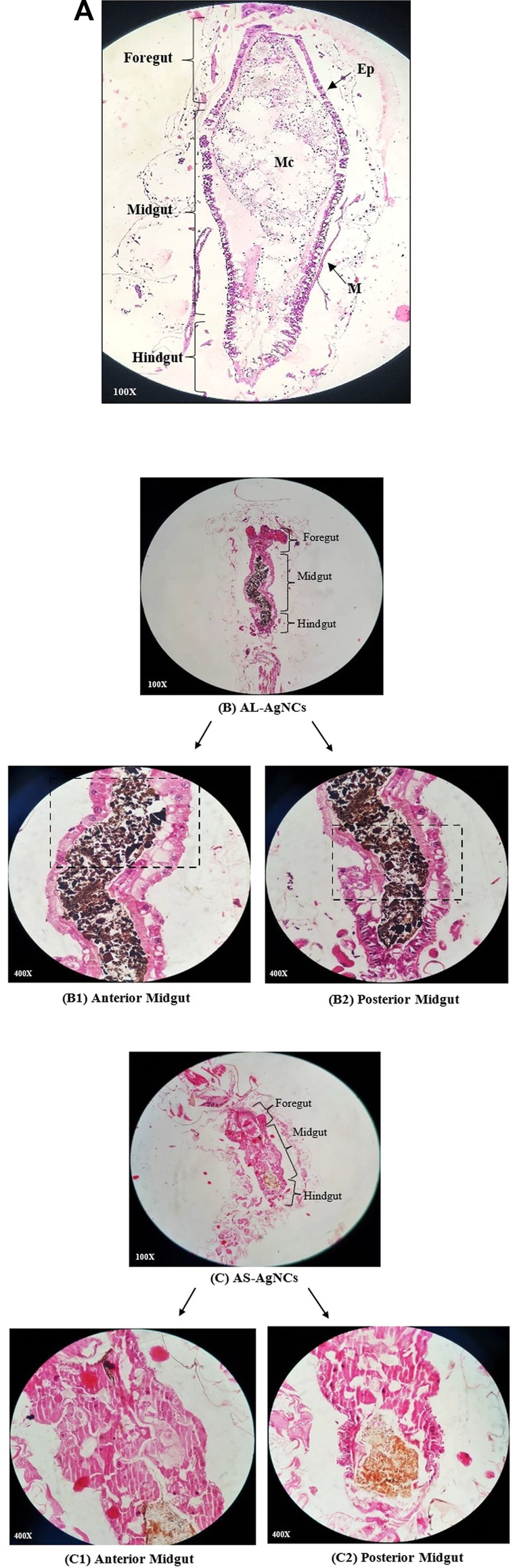
Photomicrographs of the longitudinal section of early fourth instar larvae of *Aedes aegypti* exposed for 24 h to the LC_50_ dosage of silver nanocomposites: **(A)** control, **(B)** Exposed to *Achyranthes aspera* leaf extract-mediated silver nanocomposites (AL-AgNCs), and **(C)** Exposed to *Achyranthes aspera* stem extract-mediated silver nanocomposites (AS-AgNCs).

Light microscopy of the longitudinal sections of the mid-gut of control larvae showed an unbroken and undamaged mid-gut lined evenly and entirely with epithelial cells, an intact peritrophic and basement membrane, and normal muscle forms (Figure 3A). On the other hand, the larvae exposed to AL-AgNCs and AS-AgNCs for 24 h had a relatively deformed and darkly pigmented mid-gut, containing blackened (Figure 3B) or brownish content (Figure 3C), respectively. The partial or complete destruction of mid-gut epithelial cells resulted in the widening of intercellular spaces, cytoplasmic vacuolization, destruction of the epithelial layer including nuclei and nucleoli, altered structure of microvilli, and detachment of the peritrophic membrane from the basement membrane (Figures 3B,C). These detrimental alterations in the organism’s mid-gut indicate that these changes are a conjoint response to cellular intoxication.

**FIGURE 4:**
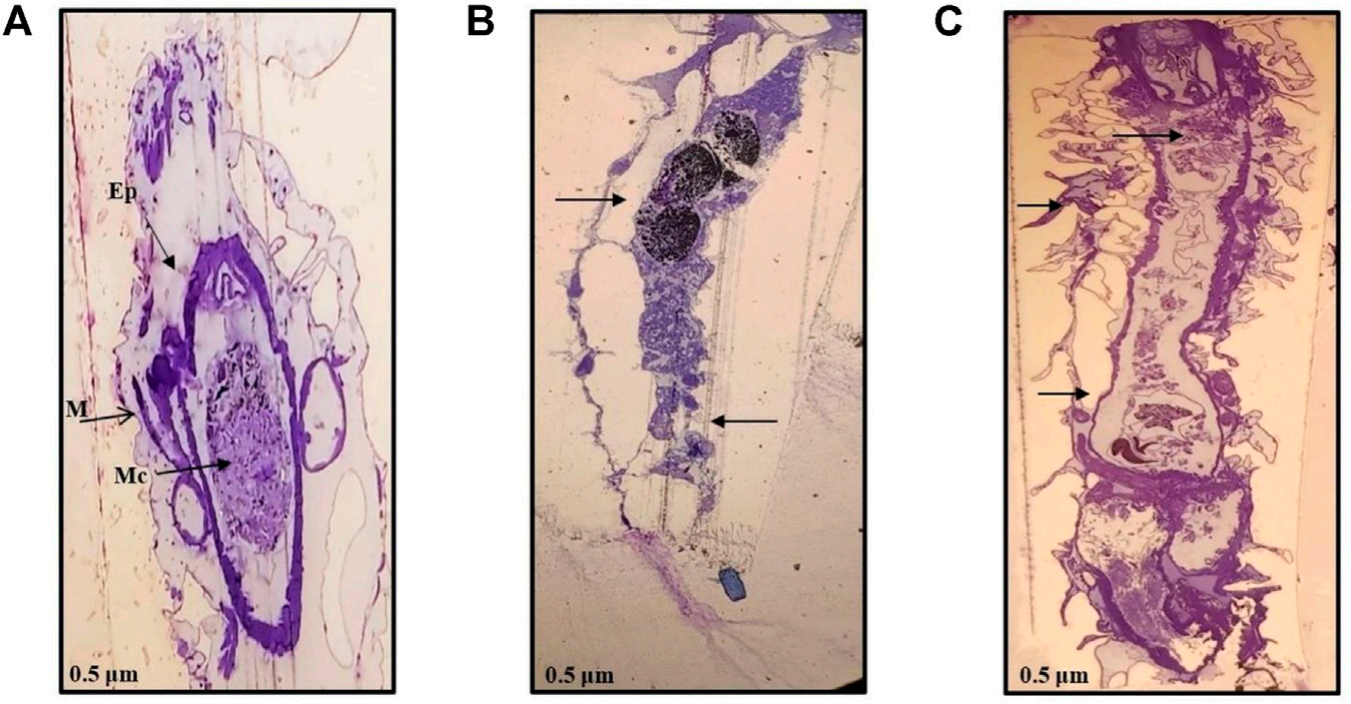
Ultra-thin histological sections of early fourth instars of *Aedes aegypti:* **(A)** control larva; **(B)** larva exposed to *Achyranthes aspera* leaf extract-mediated silver nanocomposites; **(C)** larva exposed to *Achyranthes aspera* stem extract-mediated silver nanocomposites. (→) indicates damaged area, Ep—epithelium layer, M—muscle, Mc—mid-gut content (magnification: ×100).

**FIGURE 5:**
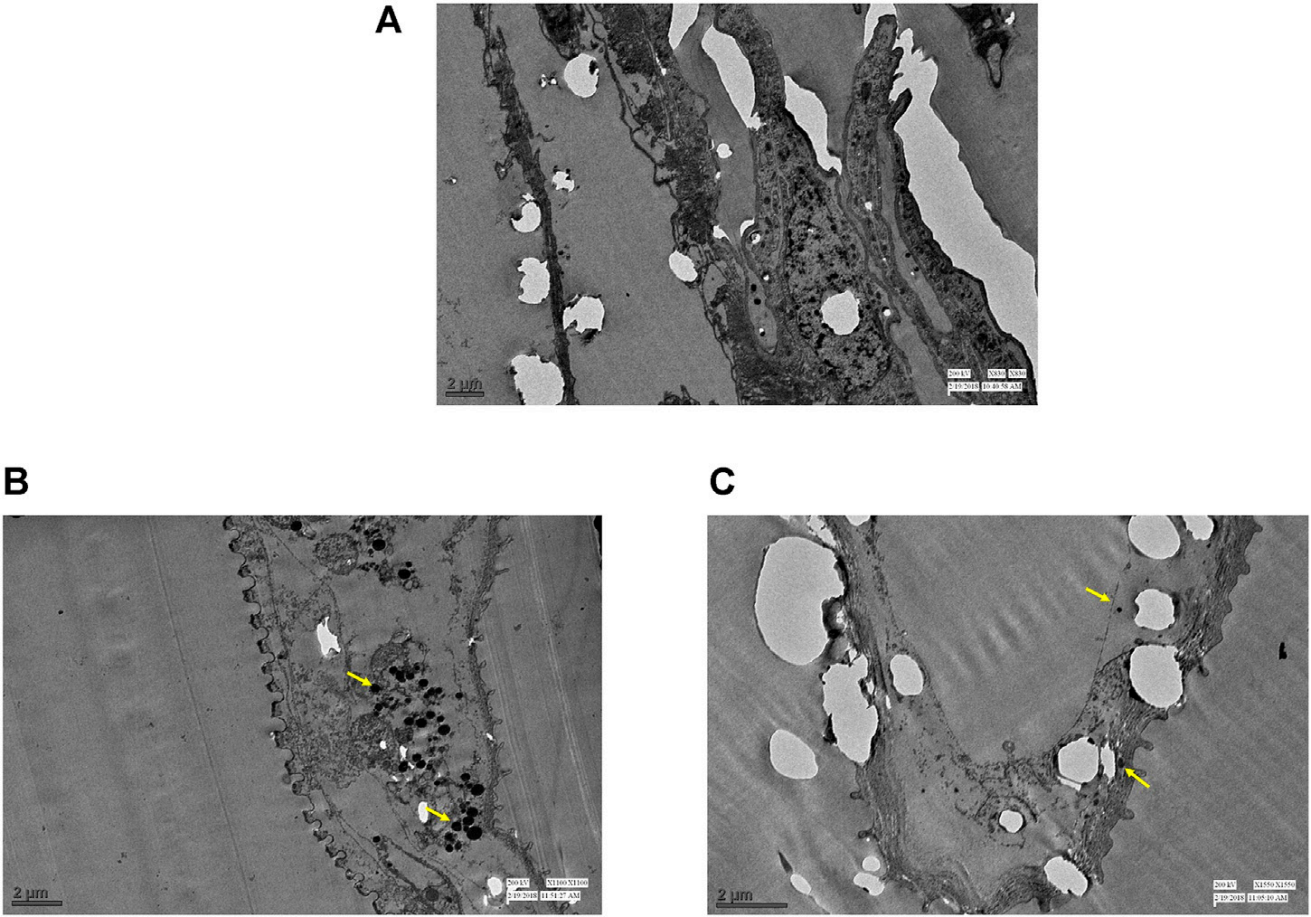
TEM images of mid-guts of early fourth instars of *Aedes aegypti*; **(A)** control; **(B)** larva exposed to *Achyranthes aspera* leaf extract-mediated silver nanocomposites (AL-AgNCs); **(C)** larva exposed to *Achyranthes aspera* stem extract-mediated silver nanocomposites (AS-AgNCs); (→) indicates deposition of black spots.

Similar observations were recorded with ultrathin-histology and transmission electron microscopy (TEM) of the mid-gut sections of the *A. aegypti* larvae. The control larvae showed a normal organization of the gut epithelium and surrounding muscles (Figures 4A, 5A). In contrast, the larvae exposed to AL-AgNCs and AS-AgNCs possessed significantly disrupted gut histoarchitecture and cellular organization (Figures 4B,C, 5B,C).

**FIGURE 6:**
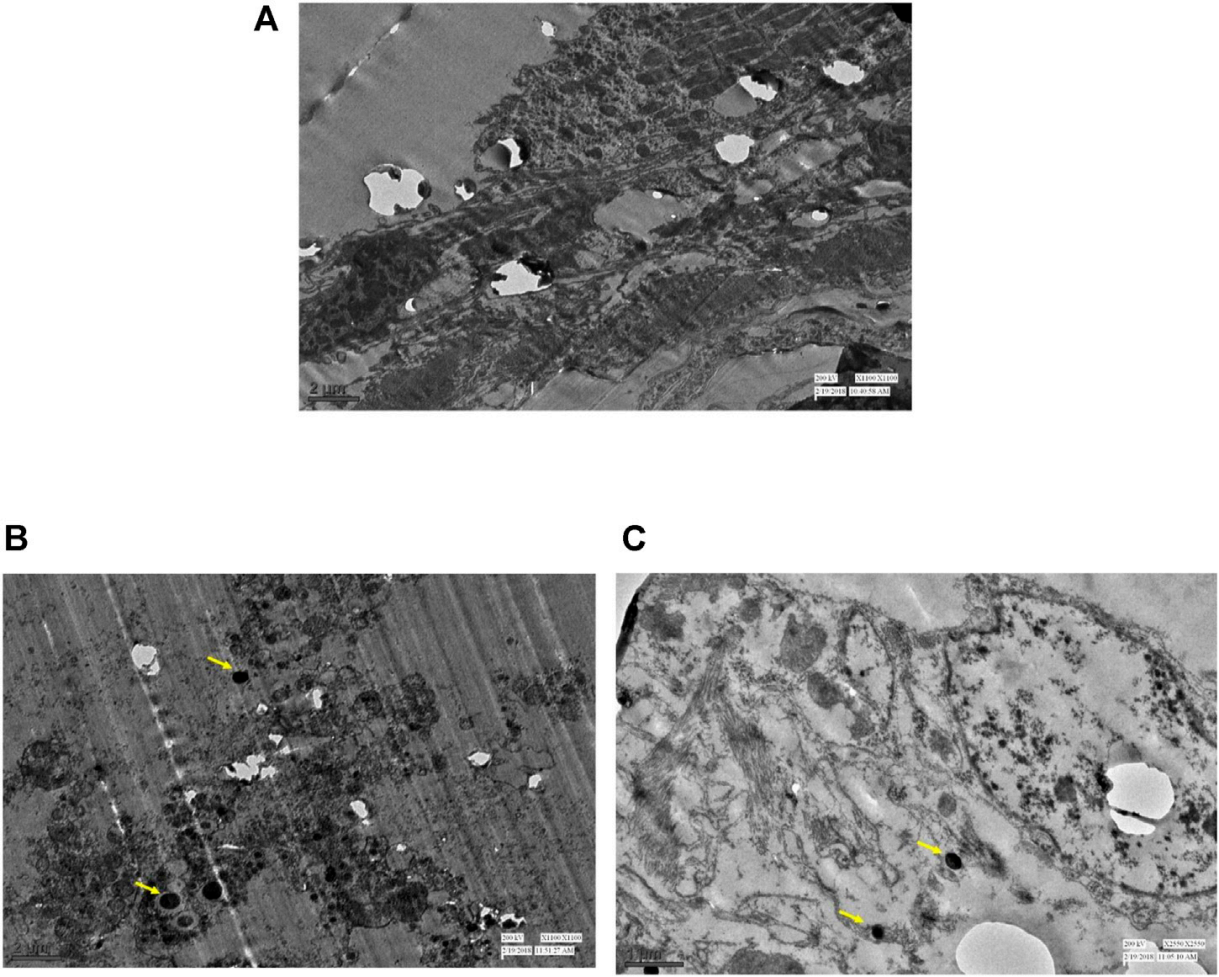
TEM images of the cellular organization of early fourth instars of *Aedes aegypti* mid-gut: **(A)** control; **(B)** larva exposed to *Achyranthes aspera* leaf extract-mediated silver nanocomposites (AL-AgNCs); **(C)** larva exposed to *Achyranthes aspera* stem extract-mediated silver nanocomposites (AS-AgNCs); and (→) indicates deposition of black spots.

The adverse impact was observed on almost all the parts of the larval mid-gut; that is, peritrophic membrane, microvilli, epithelial layer, and basement membrane. Severe damages to the cuticle membrane of the gut with a disintegrated epithelium, vacuolated cytoplasm, and desquamation of epithelial cells with intercellular spaces were noticed. The remnants of the damaged cells and body muscles were documented. The blackening of the gut content was visible. Similar observations, although more distinct, were observed with the TEM micrographs of the larval mid-gut. The control *A. aegypti* larvae showed normal cellular organization of the mid-gut architecture with intact cells (Figure 6A) as against cellular disorganization and ruptured mid-gut organization in experimental larvae (Figures 6B,C). The most significant observation was the presence of black spots (marked with yellow arrows), possibly indicating the deposition of silver particles.

## Discussion

Management of mosquitoes is of high concern nowadays because of the worldwide spread of mosquito-borne diseases. Global transmission of dengue and chikungunya has necessitated the development of an eco-friendly and efficient intervention measure which can control the *Aedes* population and diseases in the absence of an effectual vaccine and medication. Since the use of chemical insecticides has raised environmental and human health concerns, the formulation and utilization of effective nanoformulations with low toxicity to humans and high ecological safety are recommended in the management programs. The use of plant-borne compounds in the form of metallic nanocomposites is considered safe and effective against mosquitos in juvenile stages (Benelli and Mehlhorn, 2016). In light of this, attempts were undertaken to study the impact of silver nanocomposites, synthesized using *A. aspera* leaf and stem extracts, on the development, behavior, morphology, and gut histological structure of the larvae of *A. aegypti*.

The current study revealed that larval exposure to *A. aspera* nanocomposites caused dose-dependent prolonged development of larvae and decreased emergence of adults, indicating the latent effects of NCs. An enhanced effect was imparted by AS-AgNCs compared to that of the AL-AgNCs. Similar effects, causing retarded growth of larvae, have been induced by the exposure of *A. albopictus* and *A. vagus* larvae to zinc oxide nanocomposites (ZnO-NCs), suggesting the delayed effects of the NCs (Gunathilaka et al., 2021). Compared to the developmental duration in controls (8 days), they recorded an extended duration of 13 days (at LC_25_), 14.67 days (at LC_50_), 16.67 days (at LC_75_), and 17 days (at LC_100_), indicating the dose-dependent effects in the current study (Gunathilaka et al., 2021). Nevertheless, the effect of phytomediated nanocomposites has not been assessed as the modifiers of larval behavior although several studies have documented behavioral alterations in mosquito larvae on exposure to the plant extracts, revealing restlessness, excitation, coiling movements, and formation of a ring-like structure. Keeping that in mind, investigations were conducted on the impact of AA-AgNCs on the larval behavior of *A. aegypti* which did not show any noticeable behavior change except the induced mortality after 24 h. This is in contrast with the previously reported results on the effects of *A. aspera* leaf and stem extracts on *A. aegypti* larval behavior (Sharma et al., 2016) inducing aggression, convulsions, and paralysis. This indicates that although *A. aspera* crude extracts imparted effects similar to those of nerve poisons, the nanocomposites formulated with these extracts did not act as so. Thus, to estimate their plausible mode of action as contact and stomach toxicants, further investigations were conducted on the effects of NCs on the morphology and gut histology of larvae.

The morphological study of the early fourth instars of *A. aegypti* exposed to a median lethal dosage of AA-AgNCs, whether formulated with leaves or stem extracts, revealed observable effects, such as pigmentation and blackening of the gut, shrunk body, impaired and disorganized gut portion, ruptured cuticle, and a slight contraction of the internal membrane of anal papillae. These effects indicate the contact and systemic toxic effects of nanocomposites impacting both the body cuticle and gut of larvae. Our earlier studies have shown the toxic effect of these NCs on the survival of *A. aegypti* larvae (Sharma et al., 2017; Sharma et al., 2020). It is hypothesized that a damaged gut may have impaired digestion, growth, and thus, larval survival, which is also supported by the pigmented gut content induced by AA-AgNC exposure. Similar effects of *Lobelia leschenaultiana*-fabricated ZnO nanocomposites have been reported by Banumathi et al. (2017) on the external morphology of *A. aegypti* larvae. In addition, they also observed the highly shrunk abdominal region of the larvae along with the loss of antenna and mouth brush, unlike in the current study.

In order to understand the cause of pigmented gut content induced by AA-AgNCs, the current study also examined the effect of AA-AgNCs on the mid-gut histology of *A. aegypti* larvae through sectional observations. It is known that the dynamic structure of the mid-gut epithelium plays a significant role in every stage of the insect lifecycle, from beginning to end (Hixson et al., 2021). In addition, the peritrophic membrane (PM), a semipermeable and non-cellular lining of the mid-gut, protects the underlying epithelium against food abrasion, chemicals, and pathogenic attacks, and facilitates the digestive process by compartmentalization of enzymes (Gusmão et al., 2002). It has been proposed that ingested toxicants and phytochemicals may cross passively and adversely affect the insect mid-gut architecture and damage the cellular structure. Furthermore, these xenobiotics may also disrupt the PM structure, aiding their transport across the mid-gut lining and imparting toxic effects on the mid-gut. The exposure of *A. aegypti* early fourth instars to *A. aspera* leaf and stem nanocomposites induced extensive damage to the mid-gut structure leading to vacuolization, disintegration of the epithelial layer, and desquamation of epithelial cells with intercellular spaces, supporting these propositions.

The deleterious effects of NCs on the larval mid-gut indicate the high systemic toxicity of AA-AgNCs impairing the digestion, absorption, and assimilation of nutrients in larvae impacting their growth and survival. Similar effects of phytomediated NCs on the mid-gut of the *A. aegypti* larvae when exposed to *H. coronarium*-synthesized silver nanoparticles (Kalimuthu et al., 2017), *Ulva lactuca*-fabricated ZnO-NCs (Ishwarya et al., 2018), *C. serrulata-*assisted Ag, Pd, and TiO_2_ NCs (Amutha et al., 2019), and *Leonotis nepetifolia* silver nanoparticles (Manimegalai et al., 2020) have been reported. Likewise, silver nanoparticles biofabricated using essential oils of *Aquilaria sinensis* (AsEO) and *Pogostemon cablin* (PcEO) caused significant damage to the epithelial and brush border cells of the larval mid-gut of *A. albopictus* (Ga´al et al., 2018).

The current results are in corroboration with those of Sultana et al. (2020) who investigated the effects of silver nanocomposites formulated with *Cyperus rotundus* extracts on the gut histology of *A. albopictus*, *A. stephensi*, and *C. quinquefasciatus*, and observed the complete destruction of the larval gut. Similar effects of *Solanum tuberosum* carbon-dot-silver nanohybrids against *C. quinquefasciatus* larvae leading to blackening of the gut with disintegrated cellular organization and intensely damaged cuticle (Sultana et al., 2018) have been recorded. The TEM analysis of *A. aegypti* larval mid-gut exposed to the leaf extract of *Schinus terebinthifolius* displayed drastic cell disruption, disintegrated microvilli, and electron-lucent and vacuolated cytoplasm (Procópio et al., 2015). Likewise, Barua et al. (2016) put forward the fact that on the treatment of *C. quinquefasciatus* with dot–silver nanohybrid fabricated using the roots of *C. rotundus*, the hybrids entered the larval body by rupturing the cuticular membrane and subsequently destroyed the intestinal system. They suggested that cuticle rupture in the larvae lessened the membrane penetrability and affected the proton motive forces, which disturbed the intercellular functions and caused cell death.

The TEM images of *A. aegypti* larvae treated with AL-AgNCs and AS-AgNCs also showed the presence of black spots inside the mid-gut, denoting the deposition of silver particles used in the formulation of nanocomposites. These observations are in accordance with those of Sultana et al. (2018) who demonstrated the presence of silver (up to 2.98 atom%) in the *S. tuberosum-*carbon-dot-silver nanohybrid-exposed body cells of *C. quinquefasciatus* larvae through electro diffraction X-ray. Similar conclusions have been made by Sap-Iam et al. (2010) who reported shrunk larvae, penetration of polymethacrylate-stabilized AgNCs through the cuticle of *A. aegypti* larvae, and the presence of dark spots throughout the larval body indicating deposition of silver particles.

It is proposed that the small size of the AgNCs allows their passage through the insect cuticle and entry into the gut and individual cells, where they interfere with growth, molting, and other physiological processes (Govindarajan and Benelli, 2016; Sultana et al., 2020). The increased toxic and growth regulatory potential of phytomediated AgNCs may also be ascribed to their interaction with the extracellular lipoprotein matrix increasing the membrane penetrability. Similar conclusions have been made by Sundaravadivelan and Nalini (2012) who attributed the probable cause of larval death to the penetration of AgNCs through the larval membrane by interaction with cell molecules. They may probably form bonds with sulfur- or phosphorus-containing compounds present in the mid-gut, such as DNA or proteins, causing denaturation of enzymes and affecting the functions of organelles (Gnanadesigan et al., 2011; Srikar et al., 2016; Thakur et al., 2017; Sultana et al., 2018). They are also reported to generate peroxides in the mid-gut, leading to cell death (Raffi et al., 2008; Nalini et al., 2017). Another proposition is that NCs plausibly decrease the membrane permeability by reducing ion exchange and ATP synthesis leading to cell death (Sap-Iam et al., 2010).

These studies are of immense importance in the field of *Aedes* management. The field studies can ascertain the use of *A. aspera*-mediated nanocomposites in mosquito management programs.

## Conclusion

The silver nanocomposites synthesized from leaf and stem extracts of *A. aspera* adversely affected growth, morphology, and mid-gut histoarchitecture of early fourth instars of *A. aegypti.* The AgNC-exposed larvae did not show any particular change in their behavior on exposure to nanocomposites. The larvae showed shrinkage in the internal membrane of anal papillae, pigmented mid-gut, and shrunk body with an intensely damaged cuticle membrane. The mid-gut had significantly disordered internal architecture with lysed cells, damaged peritrophic membrane and microvilli, a disintegrated epithelial layer, and a ruptured and displaced basement membrane. TEM visualization of the larval mid-gut showed severe cellular damage and presence of aggregated AgNCs inside the larval body. These outcomes recommend the possible use of *A. aspera* nanocomposites as an effective and safe intervention against *A. aegypti.* Further investigations, however, would help to trace the AgNC path and understand their mode of action.

## Data Availability

The original contributions presented in the study are included in the article/Supplementary Material; further inquiries can be directed to the corresponding author.
